# A systematic review of the care coordination measurement landscape

**DOI:** 10.1186/1472-6963-13-119

**Published:** 2013-03-28

**Authors:** Ellen M Schultz, Noelle Pineda, Julia Lonhart, Sheryl M Davies, Kathryn M McDonald

**Affiliations:** 1 Center for Health Policy & Center for Primary Care and Outcomes Research, Stanford University, Stanford, CA, USA

**Keywords:** Care coordination, Quality measurement, Measure development, Systematic review

## Abstract

**Background:**

Care coordination has increasingly been recognized as an important aspect of high-quality health care delivery. Robust measures of coordination processes will be essential tools to evaluate, guide and support efforts to understand and improve coordination, yet little agreement exists among stakeholders about how to best measure care coordination. We aimed to review and characterize existing measures of care coordination processes and identify areas of high and low density to guide future measure development.

**Methods:**

We conducted a systematic review of measures published in MEDLINE through April 2012 and identified from additional key sources and informants. We characterized included measures with respect to the aspects of coordination measured (domain), measurement perspective (patient/family, health care professional, system representative), applicable settings and patient populations (by age and condition), and data used (survey, chart review, administrative claims).

**Results:**

Among the 96 included measure instruments, most relied on survey methods (88%) and measured aspects of communication (93%), in particular the transfer of information (81%). Few measured changing coordination needs (11%). Nearly half (49%) of instruments mapped to the patient/family perspective; 29% to the system representative and 27% to the health care professionals perspective. Few instruments were applicable to settings other than primary care (58%), inpatient facilities (25%), and outpatient specialty care (22%).

**Conclusions:**

New measures are needed that evaluate changing coordination needs, coordination as perceived by health care professionals, coordination in the home health setting, and for patients at the end of life.

## Background

Care coordination has increasingly been recognized as an important aspect of high-quality health care delivery, and a national priority area for improving patient care across the lifespan [1,2]. There is a growing consensus that poor coordination coupled with fragmented care compromises the quality of care patients receive and increases opportunities for negative outcomes, such as medication errors and avoidable hospitalizations and emergency department visits [1,3]. Therefore, many organizations across the health care system have undertaken quality improvement initiatives to address deficits in care coordination in order to improve patient outcomes while also containing overall health care costs.

As the interest in improving care coordination has grown, so too has the need for valid and reliable measures of care coordination. Robust measures of coordination processes will be essential tools to evaluate, guide and support efforts to understand and improve consequential deficits in care coordination. In addition to evaluating the effectiveness of improvement initiatives, such measures are important to identify deficiencies addressable by quality improvement efforts, may be used for comparative reporting or accountability and recognition purposes, and are essential for evaluating how care coordination is related to patient outcomes.

Despite the tremendous interest in evaluating and improving care coordination, key challenges remain in reaching a consensus about what constitutes care coordination, building the evidence base for care coordination, and developing measures of coordination. Due to the immature state of this field, a consensus definition of care coordination has yet to emerge. The authors of a 2007 Evidence-based Practice Center (EPC) report on care coordination identified over 40 different definitions of the term ‘care coordination’ from which they created a purposefully broad working definition of care coordination as: “The deliberate organization of patient care activities between two or more participants (including the patient) involved in a patient’s care to facilitate the appropriate delivery of health care services. Organizing care involves the marshaling of personnel and other resources needed to carry out all required patient care activities and is often managed by the exchange of information among participants responsible for different aspects of care” [4].

In addition to ambiguity about the definition of care coordination, little agreement exists among stakeholders about how to best measure it. In a review of 75 systematic reviews of care coordination interventions, the 2007 EPC report identified 20 different measures of processes related to care coordination, but none of the studies assessed the constructs of coordination directly [4].

In 2010, the Agency for Healthcare Research and Quality published the *Care Coordination Measures Atlas*, a publicly-available tool that provides details of existing care coordination measures and maps them onto a framework of perspective and coordination mechanisms [5]. The *Atlas* aims to help researchers and evaluators efficiently select instruments appropriate for their measurement needs and includes detailed information about measure format, content, reliability, validity, links to outcomes, and availability. It is available online (http://www.ahrq.gov/professionals/systems/long-term-care/resources/coordination/atlas/index.html).

This article builds on and complements the *Atlas* by providing an overview of the care coordination measurement landscape that may serve as a guide for future measure development efforts, highlighting gaps where new measures are needed and areas of high density where the field may benefit from refinement and consolidation of existing measures. We further characterized measures with respect to data, populations and settings and updated the measure search.

## Methods

We identified existing measures of care coordination processes published in the peer-reviewed literature or identified by a panel of stakeholders, and categorized those measures according to the aspects of coordination measured, patient populations to whom they have been applied, settings where they have been used, and the types of data used. This study adheres to the PRISMA guidelines for systematic reviews.

### Measure search strategy

We searched literature indexed within the MEDLINE database through July 2010 (for the *Atlas*), then updated the search through April 2012 to identify published English-language articles describing measures of care coordination using the following search strategy:

[ (“((“health care” or “healthcare” or care) adj3 (coordinat* or “co-ordinat*” or integrat*)).tw.”) AND (“(rated or rating or indicator* or measure* or valid* or reliab* or outcome* or model* or scale* or subscale* or questionnaire*).tw. or methods.fs. or exp Questionnaires/”) ] Not [ (“exp geographic locations/ not exp united states/”) ]

In addition to the literature search, we also identified measures from the 2007 EPC review of care coordination [4] and a set of ten measures recommended by the National Quality Forum [6]. To help identify additional measures, we asked a panel of 11 stakeholders, including measure developers, with expertise in care coordination and quality measurement to review our list of measures and suggest potential additions.

### Measure selection process

We reviewed titles and abstracts of literature search results to identify articles that demonstrated a relation to the measurement or evaluation of care coordination or coordinating processes. In this assessment, we relied on the working definition of care coordination proposed in the 2007 EPC review [4] and the definitions of care coordination domains from our measurement framework (Table 1).

**Table 1:** Measurement framework definitions

**Framework element**	**Definition**
**Perspectives**	
*Patient/family*	The patient or a family member completes the survey based on his/her experience.
*Health care professional*	A health care professional, or team of professionals, completes the survey or collects the measure data. Health care professionals include physicians, nurses, nurse practitioners, physician assistants, or other clinical or hospital staff.
*System representative*	A system administrator or someone else acting as a representative of a health care facility or system completes the survey, or the measure data source is from a health care delivery system, such as a medical record or administrative claims data. When an individual health care professional is providing information that reflects the system experience, rather than their individual experience, we also classify that as System Representative perspective.
**Domains**	
Coordination activities	
*Establish accountability or negotiate responsibility*	Make clear the responsibility of participants in a patient’s care for a particular aspect of that care. The accountable entity (whether a health care professional, care team, or health care organization) will be expected to answer for failures in the aspect(s) of care for which it is accountable. Specify who is primarily responsible for key care and coordination activities, the extent of that responsibility, and when that responsibility will be transferred to other care participants.
*Communicate*	Share knowledge among participants in a patient’s care. Communication may occur through a wide variety of channels, but for the purposes of measurement, we distinguish two key modes of communication: Interpersonal Communication and Information Transfer (see definitions below). While in practice interpersonal communication and information transfer often occur together, for the purposes of measurement, Interpersonal Communication is distinguished from Information Transfer by a two-way exchange of knowledge through personal interactions. Information Transfer is characterized by the transfer of data––whether orally, in writing, or electronically––and does not necessarily involve direct interaction between sender and receiver.
*Interpersonal communication*	The give-and-take of ideas, preferences, goals, and experiences through personal interactions. Examples include face-to-face interactions, telephone conversations, email, and letters.
*Information transfer*	The flow of information, such as medical history, medication lists, test results, and other clinical data, from one participant in a patient’s care to another. Examples include a written summary of laboratory results sent from a primary care practice to the patient, verbal confirmation of a laboratory value from the laboratory to a physician, or transfer of a disk containing CT images from a hospital to a primary care office.
*Facilitate transitions*	Efforts aimed at specific transitions, which occur when information about or accountability for some aspect of a patient’s care is transferred between two or more health care entities, or is maintained over time by one entity. We distinguish two types of transitions: Transitions Across Settings and Transitions As Coordination Needs Change (definitions below). All measures of transitions fit within one or both of these categories; no measures are mapped directly to the “Facilitate Transitions” heading.
*Across settings*	Examples include transitions from the inpatient (hospital) setting to the outpatient setting (e.g., physician’s offices), or transitions between ambulatory care settings (e.g., primary care to specialty clinics).
*As coordination needs change*	Examples include the transition from pediatric to adult care, transitions over the course of a woman’s changing reproductive cycle, and transitions between acute episodes of care and chronic disease management.
*Assess needs and goals*	Determine the patient’s needs for care and for coordination, including physical, emotional, and psychological health; functional status; current health and health history; self-management knowledge and behaviors; current treatment recommendations, including prescribed medications; and need for support services.
*Create a proactive plan of care*	Establish and maintain a plan of care, jointly created and managed by the patient/family and health care team, which outlines the patient’s current and longstanding needs and goals for care and/or identifies coordination gaps. The plan is designed to fill gaps in coordination, establish patient goals for care and, in some cases, set goals for the patient’s providers. Ideally, the care plan anticipates routine needs and tracks up-to-date progress toward patient goals.
*Monitor, follow-up and respond to change*	Jointly with the patient/family, assess progress toward care and coordination goals. Monitor for successes and failures in care and coordination. Refine the care plan as needed to accommodate new information or circumstances and to address any failures. Provide necessary follow-up care to patients.
*Support self-management goals*	Tailor education and support to align with patients’ capacity for and preferences about involvement in their own care. Education and support include information, training, or coaching provided to patients or their informal caregivers to promote patient understanding of and ability to carry out self-care tasks, including support for navigating their care transitions, self-efficacy, and behavior change.
*Link to community resources*	Provide information on the availability of and, if necessary, coordinate services with additional resources available in the community that may help support patients’ health and wellness or meet their care goals. Community resources are any service or program outside the health care system that may support a patient’s health and wellness. These might include financial resources (e.g., Medicaid, food stamps), social services, educational resources, schools for pediatric patients, support groups, or support programs (e.g., Meals on Wheels).
*Align resources with patient and population needs*	Within the health care setting, assess the needs of patients and populations and allocate health care resources according to those needs. At the population level, this includes developing system-level approaches to meet the needs of particular patient populations. At the patient level, it includes assessing the needs of individual patients to determine whether they might benefit from the system-level approach. For example, a system-level approach to meeting the needs of patients with cancer (the population) might be to establish a multidisciplinary tumor board meeting to help coordinate cancer care among the many relevant specialties. In this scenario, aligning a particular patient’s needs with available resources would include assessing whether that individual would likely benefit by having his/her case presented at the multidisciplinary tumor board meeting either for coordinating a consensus recommendation or for simplifying the patient’s care pathway or both.
Broad approaches potentially related to care coordination	
*Teamwork focused on coordination*	Integration among separate health care entities participating in a particular patient’s care (whether health care professionals, care teams, or health care organizations) into a cohesive and functioning whole capable of addressing patient needs.
*Health care home*	A source of usual care selected by the patient that functions as the central point for coordinating care around the patient’s needs and preferences. This includes coordination among all participants in a patient’s care, such as the patient, family members, other caregivers, primary care providers, specialists, other health care services (public and private), and nonclinical services, as needed and desired by the patient. Other terms are frequently used to describe this model, such as medical home, patient-centered medical home, and advanced primary care. Building on the work of a large and growing community, the Agency for Healthcare Research and Quality defines a medical home not simply as a place but as a model of the organization of primary care that delivers the core functions of primary health care. The medical home encompasses several functions and attributes: it is patient-centered and provides superb access to comprehensive and coordinated care and employs a system-based approach to quality and safety.
*Care management*	A process designed to assist patients and their support systems in managing their medical/social/mental health conditions more efficiently and effectively. Case management and disease management are both included in this definition.
*Medication management*	Reconciling discrepancies in medication use in order to avoid adverse drug events associated with transitions in care. This can involve review of the patient’s complete medication regimen at the time of admission/transfer/discharge, including assessing use of over-the-counter medications and supplements; comparison across information sources and settings; or direct communication between patients and providers.
*Health information technology (IT)-enabled coordination*	Tools, such as electronic medical records, patient portals, or databases, may be used to communicate information about patients and their care between health care entities (health care professionals, care teams, or health care organizations) or to maintain information over time.

Source: [5].

To this list of potential measure sources, we added any measures suggested by panelists or identified from either of the two source reports [4,6]. We then reviewed the full-text of selected articles and abstracted information about measure content, type, validity, reliability, and availability of the measure instrument. When information was lacking in a primary source, we sought additional information from the published literature, internet searches and/or the measure developer. Two reviewers used this abstracted information to exclude measures that lacked any of the following: (1) clear relevance to care coordination or any of the measurement framework domains; (2) a clearly defined and reproducible measure yielding quantitative data; and (3) information available that demonstrated valid measurement properties or information that the measure was developed in association with a logic model of theoretically-proposed or evidence-based casual linkages between the activities measured and outcomes desired. We resolved questions about measure exclusion through discussion with additional team members. In selecting and characterizing measures, we relied on measure instruments, published articles, and other supporting information gathered about each measure (e.g., user guides provided by the measure developer). Although we were particularly interested in measures applicable to ambulatory care settings, we did not limit inclusion based on health care setting.

Often, we found multiple versions of a single measure. In this article, we use the measure instrument (e.g., survey questionnaire) as the unit of analysis. Versions of a measure that varied only slightly in wording to reflect application in a different setting (e.g., primary care clinic vs. hospital) or different groups of health care providers (e.g., doctors vs. nurses) were not counted as separate instruments.

### Care coordination measurement framework

Because care coordination remains ill-defined and is potentially quite broad, we developed a framework to aid in description and classification of the measure instruments, and to structure assessment of the measurement landscape—which areas have a high density of existing measures, and which are more sparsely populated. In the absence of clear evidence about what processes are important for achieving coordinated care, the framework is based on mechanisms hypothesized to be important for achieving coordinated care and does not presume a particular quality model.

The framework, based on previous models of care coordination [7,8] and input from a second panel of 15 experts, classifies measure instruments along two dimensions: perspective and domain. Perspective refers to whose view of care coordination is being measured. It is the source of data used in the instrument: patients or family, health care professionals, or system representatives. Examples of instruments from each perspective would include a patient experience questionnaire (patient/family perspective), an audit of clinical activities completed by a nurse (health care professional perspective) and a measure calculated using administrative claims data (system representative perspective). We defined any person involved in the delivery of care to patients as a health care professional, including physicians, nurses, nurse practitioners, physicians’ assistants, or other clinical or hospital staff. We categorized any measure that used system data, such as information contained within medical records or administrative claims data, as being from the system representative perspective. We also categorized surveys within the system representative perspective when they instructed individual health care professionals to answer items while reflecting on care provided by a whole system (i.e., clinic, hospital, network). Taken together, the three perspectives are posited to offer a complete and complementary picture of an organization’s ability to coordinate care effectively and efficiently.

The second dimension we used to classify instruments is domain. Domain describes which aspects of coordination are measured. We divided the domains into two groups. The first group is Coordination Activities (12 domains), which are basic processes or actions that are hypothesized to serve as a mechanism for achieving coordination, whether employed in a systematic or improvised way. Examples include communicating, transferring information, creating a proactive plan of care, and linking to community resources. These might be thought of as processes in the Donabedian quality model [9], part of the organizational capacity in the Organizational Design model [10], or mechanisms in a context-mechanism-outcomes model [11]. The second group of domains is Broad Approaches Potentially Related to Care Coordination (5 domains). These are larger improvement efforts or care delivery models that aim to deliver high-quality care through a wide variety of means, including coordinating care. Examples of domains from this group include care management, medication management, and health care homes (also known as medical homes or patient-centered medical homes). In the Donabedian model, these might be thought of as structures, or in a Pawson model, as context. We developed and iteratively revised domain definitions with feedback from our expert panelists (Table 1).

### Measure mapping

Two members of the project team independently mapped all identified measures onto the framework of 17 domains and three measurement perspectives based on a review of the measure instruments. A single instrument could be mapped to several domains if it evaluated multiple aspects of care coordination. Likewise, a single instrument could be mapped to multiple perspectives if data were collected from different sources as part of the same instrument. We resolved disagreements through group discussion, and documented the correspondence between instrument items or elements and domains.

### Inter-rater reliability

To assess inter-rater reliability for the measure mapping, for a 10% random sample of instruments included in the original search (through July 2010) we assessed each of the three key aspects of the mapping task: (1) identifying which items from an instrument related to any of the framework domains (item mapping); (2) identifying the specific domains to which a particular item mapped (domain mapping); and (3) identifying the measurement perspective for a particular instrument (perspective mapping). We report the percent agreement for all three mapping tasks, and a kappa statistic for the item mapping. We did not calculate a kappa statistic for the domain or perspective mapping analysis because it is not an appropriate statistic when categories into which units are rated are not mutually exclusive.

### Additional measure characteristics

We also assessed the type of data (survey, chart review, administrative claims data) used by each instrument, and the patient population characteristics (age groups and conditions) and settings in which it had been applied. When an instrument focused on a particular type of transition, such as hospital to outpatient care, we mapped it to both settings. In addition to sources identified during the measure search process, when possible we also attempted to contact the developer or owner of instruments to solicit additional information about their use and asked for feedback on the patient population and setting categorizations.

## Results

### Measure search results

**Figure 1:**
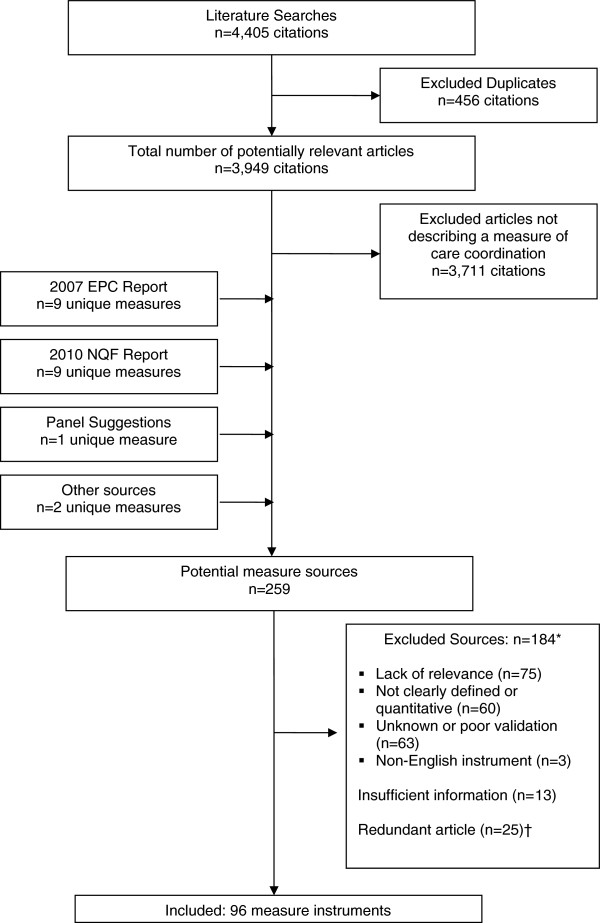
**Identification of care coordination measures.** In total, the search of MEDLINE yielded 3,949 unique articles, of which 3,711 were excluded during the initial review of titles and abstracts. An additional 21 potential measure sources were identified from other sources, including from an Evidence-based Practice Center (EPC) report on care coordination and a National Quality Forum (NQF) report on care coordination measures. The literature search and additional sources together yielded 259 potential measure sources, from which 96 unique measure instruments were included. Some measure sources yielded more than one measure. A single measure was often identified from multiple sources.*97 measure sources met more than one measure exclusion criterion. †Redundant articles are those that pertained to a measure already identified from another source.

We identified 3,949 unique, potentially relevant articles through literature searches (Figure 1), of which 3,711 were excluded after a review of titles and abstracts. We identified 21 additional potential measures through other sources. From all searches, we reviewed complete details of 259 potential measure sources, and identified 96 measure instruments for inclusion. A complete list of those instruments, their sources, and characteristics is available online (see Additional file 1).

### Reliability of measure mapping

Across six randomly selected measure instruments, there were 169 individual measure items (i.e., survey questions). Agreement about whether a specific item mapped to any of the framework domains (item mapping) was good (86%), with a kappa of 0.694 (p < 0.001). Agreement between reviewers in mapping items to each domain ranged from 80% (Communicate domain) to 100% (Facilitate Transitions as Coordination Needs Change; Health Care Home; and Health Information Technology (IT)-Enabled Coordination) across the 17 domains. For all domains, we found that reviewers agreed about 93% of 1,717 possible mappings (101 items mapped by both reviewers multiplied by 17 domains). Finally, we found that across six measures and three perspectives, reviewers agreed on all but one of the 18 possible perspective mappings, resulting in 94% agreement.

### Measurement domain and perspective

Table 2 lists the number of measure instruments mapped to each domain by perspective. Although we mapped most instruments (96%) to more than one domain, we mapped only five to more than one perspective. Those five instruments each combined patient or caregiver survey data (patient/family perspective) with administrative claims or chart review data (system representative perspective).

**Table 2:** Numbers of care coordination measure instruments, by domain and perspective

**Domain**	***Patient/family perspective***	***Health care professional perspective***	***System representative perspective***	**Total *all perspectives***
**Care coordination activities**				
Establish accountability or negotiate responsibility	22 (44%)	18 (36%)	11 (22%)	50*
Communicate	35 (58%)	17 (28%)	9 (15%)	60*
Interpersonal communication	30 (67%)	10 (22%)	5 (11%)	45
Information transfer	41 (53%)	17 (22%)	21 (27%)	78*
Facilitate transitions across settings	22 (48%)	11 (24%)	14 (30%)	46*
Facilitate transitions as coordination needs change	4 (36%)	2 (18%)	5 (45%)	11
Assess needs and goals	35 (61%)	15 (26%)	7 (12%)	57
Create a proactive plan of care	15 (36%)	15 (36%)	12 (29%)	42
Monitor, follow up, and respond to change	28 (54%)	9 (17%)	16 (31%)	52*
Support self-management goals	32 (60%)	11 (21%)	10 (19%)	53
Link to community resources	13 (46%)	8 (29%)	8 (29%)	28*
Align resources with patient and population needs	13 (43%)	8 (27%)	10 (33%)	30*
**Broad approaches potentially related to care coordination**				
Teamwork focused on coordination	16 (44%)	16 (44%)	4 (11%)	36
Health care home	8 (50%)	1 (6%)	7 (44%)	16
Care management	4 (29%)	4 (29%)	6 (43%)	14
Medication management	20 (54%)	8 (22%)	9 (24%)	37
Health IT-enabled coordination	1 (8%)	3 (23%)	9 (69%)	13

N = 96 instruments. Parentheses indicate the percent of domain instruments that map to each perspective.

*One measure instrument mapped to two perspectives for this domain, making the total less than the sum of the perspective columns.

When examining all perspectives combined, Information Transfer was measured by more included instruments (n = 78, or 81%) than was any other domain (Table 2). In addition to this domain, five others were also measured by 50% or more of the included instruments: Communicate (n = 60); Assess Needs and Goals (n = 57); Support Self-management Goals (n = 53); Monitor, Follow-up and Respond to Change (n = 52); and Establish Accountability or Negotiate Responsibility (n = 50). In contrast, only 11 instruments (11%) measured Facilitate Transitions As Coordination Needs Change, the fewest of any domain. Eight of those 11 focused on the transition from pediatric to adult care, while two measured changing needs during treatment for substance abuse and one measured changes for various phases of the lifespan.

For 12 of 17 domains, a greater percent of the instruments that mapped to the domain were measured from the patient/family perspective than from either of the other perspectives (Table 2). In contrast, no domains had more than half their measure instruments mapped to the health care professional perspective (range 6% to 44%) and only three domains had a plurality of instruments measured from the system representative perspective (Health IT-enabled Coordination; Facilitate Transitions As Needs Change, Care Management). In total, 47 instruments measured care coordination from the patient/family perspective (49%), 26 from the health care professional perspective (27%), and 28 from the system representative perspective (29%).

**Figure 2:**
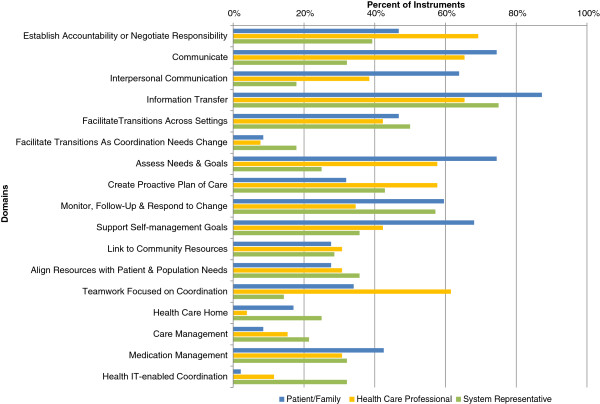
**Percent of instruments mapped to each domain, by perspective.** The percent of measure instruments that mapped to each domain are displayed separately for each of the three perspectives. Reported percentages are relative to each perspective: patient/family (n = 47 instruments, blue bars), health care professional (n = 26, orange bars) and system representative (n = 28, green bars). Five instruments mapped to multiple perspectives.

When each perspective is examined separately, several areas of measurement density become apparent (Figure 2). Most measure instruments from the patient/family perspective evaluated aspects of communication, as evidenced by the high percentage of instruments from this perspective that mapped to the Information Transfer (87%), Communicate (74%) and Interpersonal Communication (64%) domains. Indeed, nearly all patient/family perspective instruments (96%) mapped to at least one of these three communication-related domains. Instruments that measured coordination from the patient/family perspective also frequently measured the Assess Needs and Goals (74%), Support Self-Management Goals (68%) and Monitor, Follow-up and Respond to Change (60%) domains.

Instruments from the health care professional perspective also frequently measured the Information Transfer (65%) and Communicate (65%) domains, as well as the Establish Accountability or Negotiate Responsibility (69%) and Teamwork Focused on Coordination (62%) domains (Figure 2). Communication was again an area with much interest, with 23 of 26 health care professional perspective instruments (88%) measuring at least one of the three communication-related domains.

Only two areas of density stood out among the instruments mapped to the system representative perspective. As with the other perspectives, a majority of system representative instruments measured at least one of the three communication-related domains (82%); 75% measured the Information Transfer domain. Just over half (57%) of system representative perspective instruments measured the Monitor, Follow-up and Respond to Change domain (Figure 2).

Figure 2 also highlights several measurement gaps. As previously noted, few instruments measured the Facilitate Transitions As Coordination Needs Change domain from any perspective. Although frequently measured from the patient/family and health care professional perspectives, only 11% of instruments from the system representative perspective measured Interpersonal Communication or Teamwork Focused on Coordination. Few instruments from the patient/family or health care professional perspectives assessed use of health information technology to enable coordination (8% and 23%, respectively), and only one health care professional instrument measured the Health Care Home domain.

### Patient characteristics and setting

One quarter of measure instruments applied to patients of any age, while the remainder were age-specific. Just over half (51%) of included instruments were applicable to adult patients (Table 3). Of these, 20% were applicable specifically to older adults. Children were the target patient population for 22% of the instruments.

**Table 3:** Characteristics of care coordination measure instruments

**Characteristics**	**No. (%) of measure instruments**
**Patient age**	
Children	21 (22%)
Adults	49 (51%)
Older adults	19 (20%)
Not age specific	24 (25%)
Not applicable	12 (13%)
**Patient condition**	
Chronic conditions	14 (15%)
Multiple chronic conditions	7 (7%)
Cancer/Oncology	11 (11%)
Mental illness & substance use disorders	9 (9%)
Children with special health care needs	10 (10%)
End-of-life	2 (2%)
Other conditions	10 (10%)
General population/not condition-specific	47 (49%)
Not applicable	12 (13%)
**Setting**	
Inpatient care facility	24 (25%)
Emergency care facility	3 (3%)
Primary care facility	56 (58%)
Other outpatient specialty care facility	21 (22%)
Behavioral health care facility	7 (7%)
Long-term care facility	5 (5%)
Home health care	3 (3%)
Other setting*	1 (1%)
Not setting specific	28 (29%)
**Data type**	
Survey	84 (88%)
Chart review	17 (18%)
Administrative claims	14 (15%)
Other†	1 (1%)

N = 96 measure instruments. Categories within each characteristic are not mutually exclusive. Not applicable indicates that the instrument focused on health care professionals or systems, not patients.

*Habilitation services for children with disabilities in Sweden.

†Audit of coordination activities completed by nurses as care is provided.

Forty-nine percent of instruments were applicable to the general population (not condition-specific); patient condition was not applicable for a further 13% because they focused on health care professionals or systems, not patients. Of the 37 instruments that were condition-specific, 37% were targeted towards patients with chronic conditions (Table 3). Most of these instruments were intended for patients with any chronic condition, but a small number were designed for patients with a particular chronic condition, such as diabetes or HIV/AIDS. Seven instruments, or 19% of those that were condition-specific, were targeted towards patients with multiple chronic conditions. Two (5%) were focused on patients at the end of life. Among the eight instruments included in the “other conditions” category were measures used or designed for patients with cleft lip or palate, stroke, dementia, children with disabilities, and patients undergoing biopsy, cardiac or elective surgeries.

A majority of instruments (58%) were applicable to the primary care setting (Table 3) and a similar proportion (59%) were either applicable to more than one setting (n = 33) or were not setting specific (n = 28). Most (61%) of those applicable to more than one setting focused on transitions, in particular discharge from the hospital (n = 12), transitions between primary and specialty care (n = 6) and discharge from the emergency department (n = 2).

### Data types

A majority of instruments relied on survey data (88%) (Table 3), either alone (n = 76), or in combination with data collected from chart reviews (n = 4), administrative claims (n = 1), or all three data types (n = 3). Of the 17 instruments that used data from chart reviews, only one used this type as its sole data source. Similarly, all but one of the 14 measure instruments that used administrative claims data also used survey methods (n = 1), chart review (n = 9), or all 3 methods (n = 3). Many instruments used administrative claims data only to calculate a denominator, relying on information collected through chart review to generate a numerator.

## Discussion

In this study, we conducted a systematic review of care coordination measures and characterized those measures with respect to the aspects of coordination measured (domain and perspective), the settings and patient populations in which they have been applied, and the types of data used. In describing existing measures with respect to these characteristics, we aimed to highlight areas of greater and lesser measurement density to help guide further measure development work. Given care coordination’s applicability to patients at any age and most clinical scenarios, it is not surprising that the 96 measure instruments identified reflect a wide range of settings and populations.

We found the greatest density of measures to be those that assessed communication, particularly from the patient/family perspective. Other areas of density included measures of Information Transfer from all perspectives and measurement of the Assess Needs and Goals and Support Self-Management Goals domains from the patient/family perspective. Although different evaluation needs require some diversity in measure choice, a move towards greater consensus in how to measure these coordination activities is likely to help enhance the evidence base for care coordination by enabling consistent measurement across evaluations and producing data that can be more easily compared across studies. A recent publication focused on care continuity demonstrates how items from many different instruments that measure similar concepts can be developed into a new, more consolidated measure [12]. Measure endorsement bodies such as the National Quality Forum also help establish consensus in use of particular measures [13].

Although much of the interest in care coordination has focused around primary care as the “hub” of coordination, the existence of measures assessing coordination in inpatient, behavioral health, home health, and long term care settings underscores that care coordination has been recognized as important across the full care continuum.

### Measurement gaps

Although we found at least one measure that evaluated each domain from each perspective, several measurement gaps were evident from our review. We found very few measures that assessed care coordination as coordination needs change. Nearly all of those that did so focused on the transition from pediatric to adult care. Further development work would help facilitate evaluation of care coordination at other times when needs are likely to change, such as the period from an acute event to post-acute rehabilitation (e.g., following hip fracture or stroke), the change from active cancer care to ongoing surveillance, or the transition to geriatric or end-of-life care.

Health care professionals’ view of coordination was less commonly measured, suggesting that there is room for further development of these measures. Much of care coordination is performed by health care professionals, and some aspects of coordination, such as teamwork, are more readily apparent to clinicians than to patients or systems managers. For example, the use of health IT systems to enable coordination is best assessed by the clinicians who interact directly with those systems, yet we found few measures that assessed this domain from the health care professional perspective.

However, not all areas of low measure density represent a measurement gap. Some perspectives may be less applicable to particular domains. For example, the “view from above” afforded by the system representative perspective may not be well suited to evaluate interpersonal communication, which is a dynamic and local process. Similarly, patients currently may not be well situated to understand how health IT tools are used to facilitate coordination among providers or organizations, although this may change as technology, particularly use of personal health records, evolves.

We also identified some measurement gaps by setting and patient condition. We found only three measures applicable to the home health setting despite its wide use among patients likely to have high coordination needs: 10% of all stays in U.S. hospitals in 2009 were discharged to home health [14] and between 2003 and 2007, Medicare fee-for-service (FFS) beneficiaries with chronic illness had on average 6.8 home health visits during their last 6 months of life [15]. None of those three instruments were measured from the health care professional perspective, suggesting that an opportunity exists to develop additional measures of coordination specific to home health care, particularly as perceived by the providers of that care.

Another potential measurement gap relates to coordination of care at the end of life, when patients’ use of health care often intensifies. In the last six months of life, chronically ill Medicare FFS beneficiaries had on average 30.7 physician visits (13.5 with primary care physicians, 14.9 with medical specialists) and spent an average of 11.2 days in the hospital and 10.8 days in a skilled nursing facility [15], highlighting the high needs for coordination among providers in these different settings during the period of end-of-life care. Yet we found only two measures of care coordination at the end of life, both from the perspective of bereaved family members. New measures are needed that evaluate end-of-life care from the health care professional and system representative perspectives.

### Data sources

The care coordination measures we identified primarily relied on survey methods. This partially reflects the predominance among the instruments we reviewed of the patient/family perspective, which is best captured through surveys. However, use of surveys, particularly for the system representative perspective, likely also reflects the difficulty in capturing this complex, dynamic and multi-dimensional process through other data sources. Other areas of clinical quality are frequently assessed using administrative claims data, in particular hospital discharge data, and medical record review. But claims data do not capture many of the processes important to coordination, such as communication and teamwork, and information contained within medical records typically reflects care provided at only one site. Yet care coordination transcends encounters, providers and locations. The fragmentation that so often necessitates coordination also hinders its measurement by isolating information about care in silos of individual providers, records and systems. Surveys bridge these gaps by relying on the participants in a patient’s care to aggregate information across these sources, but require data collection that is often costly and time-consuming. Survey-based measures are also limited by the experience and knowledge of the individuals completing the instrument.

Interest is growing in using data from health IT systems such as electronic health records or health information exchanges to measure coordination processes. These systems hold promise in their ability to aggregate information across health care entities and facilitate information exchange, and might provide a potentially much wider selection of care coordination measures from the system representative perspective. Data from health IT systems may be particularly well-suited for measuring the Information Transfer domain, but may also provide novel insight into when, how and by whom that information is used, and how patients are connected to health care resources within their community. Several recent appraisals of the potential for measuring care coordination using health IT data suggest that advances are needed both in the technology and use of these systems before such measurement will be broadly practical [16-18]. This review found no examples of coordination measures based on health IT data.

### Limitations

As with any systematic literature review, our search strategy may have missed some relevant articles. To compensate for the limits of any single search strategy, we included three additional sources for identifying measures, including a panel of stakeholders. However, our review may omit some relevant measures, particularly those not published within the peer-reviewed literature. This review is also limited by the rapid pace of care coordination measure development, which has increased in recent years. It represents a snapshot of the measurement landscape as it stood when we concluded our measure search, and does not include newer measures.

We sought to address ambiguity about the definition of ‘care coordination’ by developing our own measurement framework and were purposefully broad in our choice of domains and perspectives, with the goal of providing information that will be useful to researchers and evaluators with many different understandings about what care coordination encompasses. Although developed based on other existing frameworks and with iterative input from our expert panelists, we recognize that our conceptualization and domain definitions may not fit with others’ understanding of care coordination. We anticipate that discussion among measure developers, stakeholders and researchers will help move the field towards a consensus definition. We also recognize that the importance of measuring the various domains included in our framework is unknown given meager evidence about which coordination activities are associated with patient and system outcomes. As the care coordination evidence base evolves, so too must this framework.

Although inter-rater reliability for measure mapping was good for all three mapping tasks, measure mapping required some judgment on the part of reviewers. Although we sought feedback from measure developers about our patient population and setting characterizations, we may have missed relevant information for some measures when developers did not respond.

Finally, we omitted several important aspects of measurement from our characterization of instruments. We examined information about feasibility whenever it was available, but ultimately did not include it among the characteristics reviewed in this study because comparable information was so often lacking from measure sources. Although we required that measures included in this review have some information available about reliability and validity, we did not attempt to systematically review or report this information. Areas with few existing measures that were identified as measurement gaps in this report might in fact have adequate coverage if one highly reliable, valid, and feasible measure is available that addresses a particular measurement need. A recent report from the Agency for Healthcare Research and Quality reports this more detailed level of analysis for a subset of *Atlas* measures with the goal of identifying instruments particularly well-suited to accountability or quality improvement evaluations of care coordination within the primary care setting [19].

## Conclusions

We identified measures of care coordination processes and characterized those measures according to a framework of domains and perspectives developed for this study. This characterization provides an overview of the care coordination measurement landscape, identifying gaps with few existing measures and areas with an abundance of measurement tools. The field may benefit if future measure development efforts focus on creating new tools to fill measurement gaps and reducing redundancy in high-density areas by refining the best of the available instruments and moving towards consensus in how to measure specific aspects of coordination from particular perspectives.

## Abbreviations

EPC: Evidence-based practice center; FFS: Fee-for-service.

## Competing interests

The authors declare they have no competing interests.

## Authors’ contributions

ES led day-to-day study management, contributed to framework development, oversaw the systematic review process, performed analyses and drafted the manuscript. NP and JL performed the systematic review, including searches and abstraction, and contributed to manuscript writing. SD provided input into interpretation of data and critical review of the manuscript. KM designed and led the study, provided input into interpretation of data and critically reviewed the manuscript. All authors read and approved the final manuscript.

## Pre-publication history

The pre-publication history for this paper can be accessed here:

http://www.biomedcentral.com/1472-6963/13/119/prepub

## Supplementary Material

Additional file 1 **Care coordination measures included within the review.** This table lists the measure instruments included within this review, including measure title, reference, perspective, number of domains measured, patient age, patient condition, setting and data types used. Click here for file
