# Supplementary material for: A systematic review of the care coordination measurement landscape
Source: BMC Health Serv Res. 2013 Mar 28;13:119. doi: 10.1186/1472-6963-13-119 (PMC3651252; doi:10.1186/1472-6963-13-119)
Supplement: Additional file 1 — Care coordination measures included within the review. This table lists the measure instruments included within this review, including measure title, reference, perspective, number of domains measured, patient age, patient condition, setting and data types used. [file 1472-6963-13-119-S1.docx]

**List of included care coordination measure instruments**

| ***Measure Title*** | ***Perspective*** | ***No. of Domains Measured****†* | ***Patient Age***‡ | ***Patient Condition***‡ | ***Setting***‡ | ***Data Type(s)*** |
| --- | --- | --- | --- | --- | --- | --- |
| Assessment of Chronic Illness Care (ACIC)*[[1](#_ENREF_1)] | System representative | 11 | Adults;  Older adults | Chronic;  Mental Illness | Primary care;  Not setting specific | Survey |
| ACOVE-2 Quality Indicators: Continuity and Coordination of Care Coordination*[[2](#_ENREF_2)] | System representative | 6 | Adults;  Older adults | Not specific | Not setting specific | Survey;  Chart Review;  Administrative |
| Coleman Measures of Care Coordination*[[3](#_ENREF_3)] | Patient/family  System representative | 6  3 | Adults;  Older adults | Chronic;  Multiple chronic | Emergency;  Outpatient specialty | Survey;  Administrative |
| Consumer Assessment of Healthcare Providers and Systems (CAHPS) Adult Primary Care 1.0*[[4](#_ENREF_4)] | Patient/family | 10 | Adults | General population | Primary care | Survey |
| CAHPS Adult Specialty Care 1.0*[[4](#_ENREF_4)] | Patient/family | 9 | Adults | General population | Outpatient specialty | Survey |
| CAHPS Child Primary Care 1.0*[[4](#_ENREF_4)] | Patient/family | 9 | Children | General population | Primary care | Survey |
| Care Coordination Measurement Tool (CCMT)*[[5](#_ENREF_5)] | Health care professional | 11 | Children | CSHCN;  Other;  General population | Primary care  Outpatient specialty | Other (audit tool) |
| Client Perception of Coordination Questionnaire (CPCQ)*[[6](#_ENREF_6)] | Patient/family | 11 | Adults;  Older adults | Chronic;  Multiple chronic;  General population | Primary care;  Outpatient specialty | Survey |
| Collaborative Practice Scale (CPS) – Nurse Scale*[[7](#_ENREF_7)] | Health care professional | 4 | Not applicable | Not applicable | Not setting specific | Survey |
| CPS - Physician Scale*[[7](#_ENREF_7)] | Health care professional | 5 | Not applicable | Not applicable | Not setting specific | Survey |
| Breast Cancer Patient and Practice Management Process Measures*[[8](#_ENREF_8)] | Health care professional | 5 | Not age specific | Cancer | Not setting specific | Survey |
| Care Transitions Measure  CTM-3*[[9](#_ENREF_9)] | Patient/family | 4 | Children;  Adults;  Older adults | General population | Inpatient;  Primary care | Survey |
| Care Transitions Measure  CTM-15*[[10](#_ENREF_10)] | Patient/family | 9 | Adults | General population | Inpatient;  Primary care;  Home health | Survey |
| Patient Assessment of Care for Chronic Conditions (PACIC)*[[11](#_ENREF_11)] | Patient/family | 9 | Adults | Chronic;  Multiple chronic | Primary care | Survey |
| Family-Centered Care Self-Assessment Tool – Family Version*[[12](#_ENREF_12)] | Patient/family | 13 | Children | CSHCN | Not setting specific | Survey |
| Family-Centered Care Self-Assessment Tool – Provider Version*[[12](#_ENREF_12)] | Health care professional | 13 | Children | CSHCN | Not setting specific | Survey |
| ICU Nurse-Physician Questionnaire – Long Version*[[13](#_ENREF_13)] | Health care professional | 8 | Not applicable | Not applicable | Inpatient | Survey |
| ICU Nurse-Physician Questionnaire – Short Version*[[13](#_ENREF_13)] | Health care professional | 6 | Not applicable | Not applicable | Inpatient | Survey |
| Primary Care Assessment Survey (PCAS)*[[14](#_ENREF_14)] | Patient/family | 8 | Adults;  Older adults | General population | Primary care | Survey |
| National Survey of Children With Special Health Care Needs (CSHCN)*[[15](#_ENREF_15)] | Patient/family | 9 | Children | CSHCN | Not setting specific | Survey |
| Head And Neck Cancer Integrated Care Indicators*[[16](#_ENREF_16)] | System representative | 4 | Adults | Cancer | Inpatient | Survey  Chart Review |
| Medical Home Index (MHI) – Long version*[[17](#_ENREF_17)] | System representative | 13 | Children;  Adults | CSHCN;  General population | Primary care | Survey |
| MHI - Short Version*[[18](#_ENREF_18)] | System representative | 8 | Children;  Adults | CSHCN;  General population | Primary care | Survey |
| Medical Home Family Index and Survey (MHFIS)*[[19](#_ENREF_19)] | Patient/family | 14 | Children | CSHCN | Primary care | Survey |
| Primary Care Assessment Tool (PCAT) – Child Expanded Edition*[[20](#_ENREF_20)] | Patient/family | 11 | Children | General population | Primary care | Survey |
| PCAT - Adult Expanded Edition*[[21](#_ENREF_21)] | Patient/family | 11 | Adults | General population | Primary care | Survey |
| PCAT - Facility Expanded Edition*[[22](#_ENREF_22)] | System representative | 12 | Not applicable | Not applicable | Primary care | Survey |
| PCAT - Provider Expanded Edition*[[22](#_ENREF_22)] | Health care professional | 12 | Not applicable | Not applicable | Primary care | Survey |
| Physician-Pharmacist Collaboration Instrument (PPCI)*[[23](#_ENREF_23)] | Health care professional | 5 | Not specific | Chronic;  General population | Primary care;  Not specific | Survey |
| Patient-Centered Medical Home (PCMH) Survey of Structural Capabilities of Primary Care Practice Sites*[[24](#_ENREF_24)] | System representative | 5 | Not applicable | Not applicable | Primary care | Survey |
| Family Medicine Medication Use Processes Matrix (MUPM)*[[25](#_ENREF_25)] | Health care professional | 7 | Not applicable | Not applicable | Primary care | Survey |
| Resources and Support for Self-Management (RSSM)*[[26](#_ENREF_26)] | Patient/family | 10 | Not specific | Chronic | Primary care | Survey |
| Continuity of Care Practices Survey – Program Level*[[27](#_ENREF_27)] | System representative | 10 | Adult | Mental illness | Behavioral health | Survey |
| CCPS - Individual Level*[[27](#_ENREF_27)] | Health care professional | 11 | Adult | Mental illness | Behavioral health | Survey |
| Nursing Home Work Environment and Performance Team Survey*[[28](#_ENREF_28)] | Health care professional | 6 | Adult;  Older adult | General population | LTC | Survey |
| Measure of Processes of Care (MPOC-28)*[[29](#_ENREF_29)] | Patient/family | 9 | Children | CSHCN;  Other | Other | Survey |
| Care Evaluation Scale for End-of-Life Care (CES)*[[30](#_ENREF_30)] | Patient/family | 5 | Not specific | Cancer;  End-of-life | Inpatient | Survey |
| Oncology Patients’ Perceptions of the Quality of Nursing Care Scale (OPPQNCS)*[[31](#_ENREF_31)] | Patient/family | 7 | Adult | Cancer;  General population | Inpatient;  Not specific | Survey |
| Care Coordination Services In Pediatric Practices*[[32](#_ENREF_32)] | Health care professional | 7 | Children | General population | Inpatient;  Primary care;  Outpatient specialty | Survey |
| Collaboration and Satisfaction About Care Decisions (CSACD)*[[33](#_ENREF_33)] | Health care professional | 2 | Not applicable | Not applicable | Inpatient | Survey |
| Follow Up Care Delivery*[[34](#_ENREF_34)] | Patient/family | 5 | Adults;  Older adults | Cancer | Primary care;  Outpatient specialty | Survey |
| Family Satisfaction in the Intensive Care Unit (FS-ICU 24)*[[35](#_ENREF_35)] | Patient/family | 4 | Adults | General population | Inpatient | Survey |
| Korean Primary Care Assessment Tool (KPCAT)*[[36](#_ENREF_36)] | Patient/family | 8 | Not specific | General population | Primary care | Survey |
| Primary Care Multimorbidity Hassles for Veterans With Chronic Illnesses*[[37](#_ENREF_37)] | Patient/family | 8 | Adults | Chronic;  Multiple chronic;  Mental illness | Primary care | Survey |
| Primary Care Satisfaction Survey for Women (PCSSW)*[[38](#_ENREF_38)] | Patient/family | 7 | Adults | General population | Primary care | Survey |
| Personal Health Records (PHR)*[[39](#_ENREF_39)] | System representative | 4 | Not applicable | Not applicable | Not specific | Survey |
| Picker Patient Experience (PPE-15)*[[40](#_ENREF_40)] | Patient/family | 6 | Adults | General population | Inpatient | Survey |
| Physician Office Quality of Care Monitor (QCM)*[[41](#_ENREF_41)] | Patient/family | 6 | Not specific | General population | Primary care;  Outpatient specialty | Survey |
| Patient Perceptions of Care (PPOC)*[[42](#_ENREF_42)] | Patient/family | 10 | Adults | General population | Primary care;  Outpatient specialty;  Behavioral health | Survey |
| PREPARED Survey – Patient version*[[43](#_ENREF_43)] | Patient/family | 9 | Adults;  Older adults | General population | Inpatient;  Primary care | Survey |
| PREPARED Survey – Carer Version[[43](#_ENREF_43)] | Patient/family | 8 | Adults;  Older adults | General population | Inpatient;  Primary care | Survey |
| PREPARED Survey – Residential Care Staff Version*[[44](#_ENREF_44)] | Health care professional | 4 | Adults;  Older adults | General population | Inpatient;  Primary care;  LTC | Survey |
| PREPARED Survey – Community Service Provider Version*[[44](#_ENREF_44)] | Health care professional | 6 | Adults;  Older adults | General population | Inpatient;  LTC;  Not specific | Survey |
| PREPARED Survey – Medical Practitioner Version*[[43](#_ENREF_43)] | Health care professional | 10 | Adults;  Older adults | General population | Inpatient;  Primary care | Survey |
| PREPARED Survey – Modified Medical Practitioner Version*[[45](#_ENREF_45)] | Health care professional | 7 | Adults | General population | Inpatient;  Primary care | Survey |
| Health Tracking Household Survey*[[46](#_ENREF_46)] | Patient/family | 4 | Adults | Chronic;  General population | Primary care;  Outpatient specialty | Survey |
| Adapted Picker Institute Cancer Survey*[[47](#_ENREF_47)] | Patient/family | 9 | Adults | Cancer | Inpatient | Survey |
| Ambulatory Care Experiences Survey (ACES)*[[48](#_ENREF_48)] | Patient/family | 5 | Children;  Adults | General population | Primary care | Survey |
| Patient Perception of Continuity Instrument (PC)*[[49](#_ENREF_49)] | Patient/family | 6 | Adults | General population | Primary care | Survey |
| Jefferson Survey of Attitudes Toward Physician-Nurse Collaboration*[[50](#_ENREF_50)] | Health care professional | 6 | Not applicable | Not applicable | Not specific | Survey |
| Clinical Microsystem Assessment Tool (CMAT)*[[51](#_ENREF_51)] | System representative | 6 | Not applicable | Not applicable | Not specific | Survey |
| Components of Primary Care Index (CPCI)*[[52](#_ENREF_52)] | Patient/family | 7 | Children;  Adults;  Not specific | General population | Primary care;  Outpatient specialty | Survey |
| Relational Coordination Survey*[[53](#_ENREF_53)] | Health care professional | 4 | Adults;  Older adults;  Not specific | Chronic;  Multiple chronic;  Other;  General population | Inpatient;  Primary care;  Outpatient specialty;  LTC | Survey |
| Fragmentation of Care Index (FCI)*[[54](#_ENREF_54)] | System representative | 1 | Adults | Chronic;  Multiple chronic | Primary care;  Outpatient specialty | Chart review |
| After-Death Bereaved Family Member Interview*[[55](#_ENREF_55)] | Patient/family | 5 | Adults;  Older adults | End-of-Life | Inpatient;  LTC;  Home health | Survey |
| Schizophrenia Quality Indicators for Integrated Care*[[56](#_ENREF_56)] | Patient/family  System representative | 1  8 | Not specific | Mental illness | Not specific | Survey  Chart review  Administrative |
| Degree of Clinical Integration Measures*[[57](#_ENREF_57)] | System representative | 3 | Not specific | General population | Not specific | Chart review  Administrative |
| National Survey for Children’s Health (NSCH)*[[58](#_ENREF_58)] | Patient/family | 7 | Children | General population | Not specific | Survey |
| Mental Health Professional HIV/AIDS Point Prevalence and Treatment Experiences Survey  Part II*[[59](#_ENREF_59)] | System representative | 6 | Adults | Chronic;  Multiple chronic;  Mental illness | Primary care;  Behavioral health | Survey |
| Cardiac Rehabilitation Patient Referral from an Inpatient Setting*[[60](#_ENREF_60)] | System representative | 1 | Adults;  Not specific | Chronic;  Other | Inpatient;  Outpatient specialty | Chart review  Administrative |
| Cardiac Rehabilitation Patient Referral from an Outpatient Setting*[[60](#_ENREF_60)] | System representative | 2 | Adults;  Not specific | Chronic;  Other | Outpatient specialty;  Not specific | Chart review  Administrative |
| Patients with a Transient Ischemic Event ER Visit That Had a Follow Up Office Visit*[[61](#_ENREF_61)] | System representative | 2 | Adults | Other | Emergency;  Primary care;  Outpatient specialty | Administrative |
| Biopsy Follow Up*[[61](#_ENREF_61)] | System representative | 1 | Not specific | Other | Primary care;  Not specific | Chart review  Administrative |
| Reconciled Medication List Received by Discharged Patients*[[62](#_ENREF_62)] | System representative | 4 | Not specific | General population | Inpatient;  Primary care;  Not specific | Chart review  Administrative |
| Transition Record with Specified Elements Received by Discharged Patients (Inpatient Discharges)*[[62](#_ENREF_62)] | System representative | 6 | Not specific | General population | Inpatient;  Primary care;  Not specific | Chart review  Administrative |
| Timely Transmission of Transition Record*[[62](#_ENREF_62)] | System representative | 5 | Not specific | General population | Inpatient;  Primary care;  Not specific | Chart review  Administrative |
| Transition Record with Specified Elements Received by Discharged Patients (Emergency Department Discharges)*[[62](#_ENREF_62)] | System representative | 6 | Not specific | General population | Emergency;  Primary care;  Outpatient specialty;  Home health | Chart review  Administrative |
| Melanoma Continuity of Care—Recall System*[[63](#_ENREF_63)] | System representative | 1 | Not specific | Cancer | Not specific | Chart review  Administrative |
| Team Survey for Program of All-inclusive Care for the Elderly (PACE)[[64](#_ENREF_64)] | Health care professional | 5 | Adults;  Older adults | General population | Primary care;  Outpatient specialty | Survey |
| Patient Experiences with Cancer Care Survey[[65](#_ENREF_65)] | Patient/family | 4 | Adults | Cancer | Not specific | Survey |
| Parents' Attitudes Towards Communication of Physicians and Complementary and Alternative Medicine (CAM) Practitioners[[66](#_ENREF_66)] | Patient/family | 8 | Children | General population | Primary care;  Outpatient specialty | Survey |
| Multiple Sclerosis Quality Indicators[[67](#_ENREF_67)] | Patient/family  System representative | 1  6 | Not specific | Chronic | Not specific | Survey  Chart review  Administrative |
| Integrated Healthcare Model Study 2: Primary Care Provider Questionnaire[[68](#_ENREF_68)] | Health care professional | 6 | Not specific | Mental illness | Primary care;  Behavioral health | Survey |
| Integrated Healthcare Model Study 2: Behavioral Healthcare Provider Questionnaire[[68](#_ENREF_68)] | Health care professional | 9 | Not specific | Mental illness | Primary care;  Behavioral health | Survey |
| Breast Cancer Patients' Perceptions of Care Coordination[[69](#_ENREF_69)] | Patient/family | 5 | Adults | Cancer | Not specific | Survey |
| Medical Care Questionnaire (MCQ)[[70](#_ENREF_70)] | Patient/family | 2 | Not specific | Cancer | Outpatient specialty | Survey |
| Community Mental Health Teams for Older People (CMHTsOP) Team Integration Indicators[[71](#_ENREF_71)] | Health care professional | 4 | Adults;  Older adults | Mental illness | Behavioral health | Survey |
| The Joint Commission Patient-Centered Medical Home Self-assessment Survey | System representative | 15 | Not specific | General population | Primary care;  Outpatient specialty | Survey |
| Collaborative Care for Attention-Deficit Disorder Scale (CCADDS)[[73](#_ENREF_73)] | Health care professional | 10 | Children | CSHCN | Primary care | Survey |
| Follow-up Coordination Survey[[74](#_ENREF_74)] | Health care professional | 3 | Children | General population | Primary care | Survey  Chart review |
| CAHPS Patient-Centered Medical Home Supplementary Survey[[75](#_ENREF_75)] | Patient/family | 3 | Children;  Adults | General population | Primary care;  Outpatient specialty | Survey |
| Parents' Perceptions of Primary Care (P3C)[[76](#_ENREF_76)] | Patient/family | 5 | Children | General population | Primary care | Survey |
| Patient Satisfaction with Cancer-related Care (PSCC)[[77](#_ENREF_77)] | Patient/family | 4 | Adults | Cancer | Not specific | Survey |
| Primary Care Questionnaire for Complex Pediatric Patients[[78](#_ENREF_78)] | Patient/family  System representative | 7  11 | Children | CSHCN | Primary care | Survey  Chart review |
| Dementia Guideline-Derived Quality of Care Indicators[[79](#_ENREF_79)] | Patient/family  System representative | 5  3 | Adults;  Older adults | Other | Not specific | Survey  Chart review |
| Canadian Survey of Experiences with Primary Health Care Questionnaire[[80](#_ENREF_80)] | Patient/family | 11 | Not specific | General population | Primary care | Survey |

*Further details of this measure, including the domains to which it maps, are included in the measure profile within the *Care Coordination Measures Atlas* [[81](#_ENREF_81)]

†When more than one perspective is measured by a single instrument, the number of domains measured is reported separately for each perspective.

‡Characterization based on measure intent or any known use. Some measures have been used in multiple studies, and therefore may be mapped to more than one specific category in addition to a non-specific category.

CSHCN – Children with special health care needs; LTC – Long-term care

**References**

1. Bonomi AE, Wagner EH, Glasgow RE, VonKorff M: **Assessment of chronic illness care (ACIC): a practical tool to measure quality improvement**. *Health Serv Res* 2002, **37**(3):791-820.

2. Wenger NS, Young RT: **Quality indicators for continuity and coordination of care in vulnerable elders**. *J Am Geriatr Soc* 2007, **55 Suppl 2**:S285-292.

3. Coleman EA, Eilertsen TB, Magid DJ, Conner DA, Beck A, Kramer AM: **The association between care co-ordination and emergency department use in older managed care enrollees**. *Int J Integr Care* 2002, **2**:e03.

4. **CAHPS Surveys and Tools** [https://[www.cahps.ahrq.gov/default.asp](http://www.cahps.ahrq.gov/default.asp)]

5. Antonelli RC, Antonelli DM: **Providing a medical home: the cost of care coordination services in a community-based, general pediatric practice**. *Pediatrics* 2004, **113**(5 Suppl):1522-1528.

6. McGuiness C, Sibthorpe B: **Development and initial validation of a measure of coordination of health care**. *Int J Qual Health Care* 2003, **15**(4):309-318.

7. Weiss SJ, Davis HP: **Validity and reliability of the Collaborative Practice Scales**. *Nurs Res* 1985, **34**(5):299-305.

8. Katz SJ, Hawley ST, Morrow M, Griggs JJ, Jagsi R, Hamilton AS, Graff JJ, Friese CR, Hofer TP: **Coordinating cancer care: patient and practice management processes among surgeons who treat breast cancer**. *Med Care* 2010, **48**(1):45-51.

9. Parry C, Mahoney E, Chalmers SA, Coleman EA: **Assessing the quality of transitional care: further applications of the care transitions measure**. *Med Care* 2008, **46**(3):317-322.

10. Coleman EA, Smith JD, Frank JC, Eilertsen TB, Thiare JN, Kramer AM: **Development and testing of a measure designed to assess the quality of care transitions**. *Int J Integr Care* 2002, **2**:e02.

11. Glasgow RE, Wagner EH, Schaefer J, Mahoney LD, Reid RJ, Greene SM: **Development and validation of the Patient Assessment of Chronic Illness Care (PACIC)**. *Med Care* 2005, **43**(5):436-444.

12. **Family Centered Care Self-Assessment Tool - Family Version. October 2008.** [<http://www.familyvoices.org/pub/index.php?topic=fcc>]

13. Shortell SM, Rousseau DM, Gillies RR, Devers KJ, Simons TL: **Organizational assessment in intensive care units (ICUs): construct development, reliability, and validity of the ICU nurse-physician questionnaire**. *Med Care* 1991, **29**(8):709-726.

14. Safran DG, Kosinski M, Tarlov AR, Rogers WH, Taira DH, Lieberman N, Ware JE: **The Primary Care Assessment Survey: tests of data quality and measurement performance**. *Med Care* 1998, **36**(5):728-739.

15. Blumberg SJ, Welch EM, Chowdhury SR, Upchurch HL, Parker EK, Skalland BJ: **Design and operation of the National Survey of Children with Special Health Care Needs, 2005-2006**. *Vital and health statistics Ser 1, Programs and collection procedures* 2008(45):1-188.

16. Ouwens MM, Marres HA, Hermens RR, Hulscher MM, van den Hoogen FJ, Grol RP, Wollersheim HC: **Quality of integrated care for patients with head and neck cancer: Development and measurement of clinical indicators**. *Head & neck* 2007, **29**(4):378-386.

17. Cooley WC, McAllister JW, Sherrieb K, Clark RE: **The Medical Home Index: Development and validation of a new practice-level measure of implementation of the Medical Home model**. *Ambulatory Pediatrics* 2003, **3**(4):173-180.

18. **Center for Medical Home Improvement** [<http://www.medicalhomeimprovement.org/knowledge/practices.html#measurement>]

19. McAllister JW, Sherrieb K, Cooley WC: **Improvement in the Family-Centered Medical Home Enhances Outcomes for Children and Youth with Special Healthcare Needs**. *Journal of Ambulatory Care Management* 2009, **32**(3):188-196.

20. Cassady CE, Starfield B, Hurtado MP, Berk RA, Nanda JP, Friedenberg LA: **Measuring consumer experiences with primary care**. *Pediatrics* 2000, **105**(4):998-1003.

21. Shi L: **Validating the Adult Primary Care Assessment Tool**. *J Fam Pract* 2001, **50**:161.

22. **Johns Hopkins University Bloomberg School of Public Health Primary Care Assessment Tools** [<http://www.jhsph.edu/pcpc/pca_tools.html>]

23. Zillich AJ, Doucette WR, Carter BL, Kreiter CD: **Development and initial validation of an instrument to measure physician-pharmacist collaboration from the physician perspective**. *Value in health : the journal of the International Society for Pharmacoeconomics and Outcomes Research* 2005, **8**(1):59-66.

24. Friedberg MW, Safran DG, Coltin KL, Dresser M, Schneider EC: **Readiness for the Patient-Centered Medical Home: structural capabilities of Massachusetts primary care practices**. *J Gen Intern Med* 2009, **24**(2):162-169.

25. Farrell B, Pottie K, Woodend K, Yao VH, Kennie N, Sellors C, Martin C, Dolovich L: **Developing a tool to measure contributions to medication-related processes in family practice**. *Journal of interprofessional care* 2008, **22**(1):17-29.

26. McCormack LA, Williams-Piehota PA, Bann CM, Burton J, Kamerow DB, Squire C, Fisher E, Brownson CA, Glasgow RE: **Development and validation of an instrument to measure resources and support for chronic illness self-management: a model using diabetes**. *Diabetes Educ* 2008, **34**(4):707-718.

27. Schaefer JA, Cronkite R, Ingudomnukul E: **Assessing continuity of care practices in substance use disorder treatment programs**. *Journal of studies on alcohol* 2004, **65**(4):513-520.

28. Temkin-Greener H, Zheng N, Katz P, Zhao H, Mukamel DB: **Measuring work environment and performance in nursing homes**. *Med Care* 2009, **47**(4):482-491.

29. Granat T, Lagander B, Borjesson MC: **Parental participation in the habilitation process--evaluation from a user perspective**. *Child Care Health Dev* 2002, **28**(6):459-467.

30. Morita T, Hirai K, Sakaguchi Y, Maeyama E, Tsuneto S, Shima Y: **Measuring the quality of structure and process in end-of-life care from the bereaved family perspective**. *J Pain Symptom Manage* 2004, **27**(6):492-501.

31. Radwin LE, Cabral HJ, Wilkes G: **Relationships between patient-centered cancer nursing interventions and desired health outcomes in the context of the health care system**. *Res Nurs Health* 2009, **32**(1):4-17.

32. Gupta G, Unruh ML, Nolin TD, Hasley PB: **Primary care of the renal transplant patient**. *Journal of General Internal Medicine* 2010, **25**(7):731-740.

33. Baggs JG: **Development of an instrument to measure collaboration and satisfaction about care decisions**. *J Adv Nurs* 1994, **20**(1):176-182.

34. Haggstrom DA, Arora NK, Helft P, Clayman ML, Oakley-Girvan I: **Follow-up care delivery among colorectal cancer survivors most often seen by primary and subspecialty care physicians**. *J Gen Intern Med* 2009, **24 Suppl 2**:S472-479.

35. Stricker KH, Kimberger O, Schmidlin K, Zwahlen M, Mohr U, Rothen HU: **Family satisfaction in the intensive care unit: what makes the difference?** *Intensive Care Med* 2009, **35**(12):2051-2059.

36. Lee JH, Choi YJ, Sung NJ, Kim SY, Chung SH, Kim J, Jeon TH, Park HK, Korean Primary Care Research G: **Development of the Korean primary care assessment tool--measuring user experience: tests of data quality and measurement performance**. *Int J Qual Health Care* 2009, **21**(2):103-111.

37. Parchman ML, Noel PH, Lee S: **Primary care attributes, health care system hassles, and chronic illness**. *Med Care* 2005, **43**(11):1123-1129.

38. Scholle SH, Weisman CS, Anderson RT, Camacho F: **The development and validation of the primary care satisfaction survey for women**. *Womens Health Issues* 2004, **14**(2):35-50.

39. Reti SR, Feldman HJ, Ross SE, Safran C: **Improving personal health records for patient-centered care**. *J Am Med Inform Assoc* 2010, **17**(2):192-195.

40. Jenkinson C, Coulter A, Bruster S: **The Picker Patient Experience Questionnaire: development and validation using data from in-patient surveys in five countries**. *Int J Qual Health Care* 2002, **14**(5):353-358.

41. Seibert JH, Strohmeyer JM, Carey RG: **Evaluating the physician office visit: in pursuit of a valid and reliable measure of quality improvement efforts**. *The Journal of ambulatory care management* 1996, **19**(1):17-37.

42. Borowsky SJ, Nelson DB, Fortney JC, Hedeen AN, Bradley JL, Chapko MK: **VA community-based outpatient clinics: performance measures based on patient perceptions of care**. *Med Care* 2002, **40**(7):578-586.

43. Grimmer K, Moss J: **The development, validity and application of a new instrument to assess the quality of discharge planning activities from the community perspective**. *Int J Qual Health Care* 2001, **13**(2):109-116.

44. **International Centre for Allied Health Evidence** [<http://www.unisa.edu.au/cahe/Resources/DCP/Information.asp>]

45. Graumlich JF, Grimmer-Somers K, Aldag JC: **Discharge planning scale: community physicians' perspective**. *J Hosp Med* 2008, **3**(6):455-464.

46. O'Malley AS, Cunningham PJ: **Patient experiences with coordination of care: the benefit of continuity and primary care physician as referral source**. *J Gen Intern Med* 2009, **24**(2):170-177.

47. Ayanian JZ, Zaslavsky AM, Guadagnoli E, Fuchs CS, Yost KJ, Creech CM, Cress RD, O'Connor LC, West DW, Wright WE: **Patients' perceptions of quality of care for colorectal cancer by race, ethnicity, and language**. *Journal of clinical oncology : official journal of the American Society of Clinical Oncology* 2005, **23**(27):6576-6586.

48. Safran DG, Karp M, Coltin K, Chang H, Li A, Ogren J, Rogers WH: **Measuring patients' experiences with individual primary care physicians. Results of a statewide demonstration project**. *J Gen Intern Med* 2006, **21**(1):13-21.

49. Chao J: **Continuity of care: incorporating patient perceptions**. *Fam Med* 1988, **20**(5):333-337.

50. Hojat M, Fields SK, Veloski JJ, Griffiths M, Cohen MJ, Plumb JD: **Psychometric properties of an attitude scale measuring physician-nurse collaboration**. *Evaluation & the health professions* 1999, **22**(2):208-220.

51. **Intitute for Healthcare Improvement** [<http://www.ihi.org/IHI/Topics/Improvement/ImprovementMethods/Tools/ClinicalMicrosystemAssessmentTool.htm>]

52. Flocke SA: **Measuring attributes of primary care: development of a new instrument**. *J Fam Pract* 1997, **45**(1):64-74.

53. Gittell JH, Fairfield KM, Bierbaum B, Head W, Jackson R, Kelly M, Laskin R, Lipson S, Siliski J, Thornhill T *et al*: **Impact of relational coordination on quality of care, postoperative pain and functioning, and length of stay: a nine-hospital study of surgical patients**. *Med Care* 2000, **38**(8):807-819.

54. Liu CW, Einstadter D, Cebul RD: **Care fragmentation and emergency department use among complex patients with diabetes**. *Am J Manag Care* 2010, **16**(6):413-420.

55. Teno JM, Clarridge B, Casey V, Edgman-Levitan S, Fowler J: **Validation of Toolkit After-Death Bereaved Family Member Interview**. *J Pain Symptom Manage* 2001, **22**(3):752-758.

56. Weinmann S, Roick C, Martin L, Willich S, Becker T: **Development of a set of schizophrenia quality indicators for integrated care**. *Epidemiologia e psichiatria sociale* 2010, **19**(1):52-62.

57. Devers KJ, Shortell SM, Gillies RR, Anderson DA, Mitchell JB, Erickson KL: **Implementing organized delivery systems: an integration scorecard**. *Health Care Manage Rev* 1994, **19**(3):7-20.

58. **National Survey of Children's Health Data Resource Center for Child and Adolescent Health** [<http://www.nschdata.org/content/Default.aspx>]

59. Lemmon R, Shuff IM: **Effects of mental health centre staff turnover on HIV/AIDS service delivery integration**. *AIDS care* 2001, **13**(5):651-661.

60. American Association of Cardiovascular and Pulmonary Rehabilitation, American College of Cardiology Foundation, American Heart Association Task Force on Performance Measures, Thomas RJ, King M, Lui K, Oldridge N, Pina IL, Spertus J: **AACVPR/ACCF/AHA 2010 Update: Performance Measures on Cardiac Rehabilitation for Referral to Cardiac Rehabilitation/Secondary Prevention Services Endorsed by the American College of Chest Physicians, the American College of Sports Medicine, the American Physical Therapy Association, the Canadian Association of Cardiac Rehabilitation, the Clinical Exercise Physiology Association, the European Association for Cardiovascular Prevention and Rehabilitation, the Inter-American Heart Foundation, the National Association of Clinical Nurse Specialists, the Preventive Cardiovascular Nurses Association, and the Society of Thoracic Surgeons**. *Journal of the American College of Cardiology* 2010, **56**(14):1159-1167.

61. National Quality Forum: **Preferred Practices and Performance Measures for Measuring and Reporting Care Coordination: A Consensus Report**. Washington, DC: National Quality Forum; 2010.

62. American Board of Internal Medicine Foundation, American College of Physicians, Society of Hospital Medicine, Physician Consortium for Performance Improvement: **Care Transitions Performance Measurement Set (Phase I: Inpatient discharges and emergency department discharges)**. Chicago, IL: American Medical Association; 2009.

63. American Academy of Dermatology, Physician Consortium for Performance Improvement, National Committee for Quality Assurance: **Melanoma II Physician Performance Measurement Set**. Chicago, IL and Washington, D.C.: American Medical Association and National Committee for Quality Assurance; 2007.

64. Temkin-Greener H, Gross D, Kunitz SJ, Mukamel D: **Measuring interdisciplinary team performance in a long-term care setting**. *Med Care* 2004, **42**(5):472-481.

65. Ayanian JZ, Zaslavsky AM, Arora NK, Kahn KL, Malin JL, Ganz PA, van Ryn M, Hornbrook MC, Kiefe CI, He Y *et al*: **Patients' experiences with care for lung cancer and colorectal cancer: findings from the Cancer Care Outcomes Research and Surveillance Consortium**. *Journal of clinical oncology : official journal of the American Society of Clinical Oncology* 2010, **28**(27):4154-4161.

66. Ben-Arye E, Traube Z, Schachter L, Haimi M, Levy M, Schiff E, Lev E: **Integrative pediatric care: parents' attitudes toward communication of physicians and CAM practitioners**. *Pediatrics* 2011, **127**(1):e84-95.

67. Cheng EM, Crandall CJ, Bever CT, Jr., Giesser B, Haselkorn JK, Hays RD, Shekelle P, Vickrey BG: **Quality indicators for multiple sclerosis**. *Multiple sclerosis* 2010, **16**(8):970-980.

68. Funderburk JS, Sugarman DE, Maisto SA, Ouimette P, Schohn M, Lantinga L, Wray L, Batki S, Nelson B, Coolhart D *et al*: **The description and evaluation of the implementation of an integrated healthcare model**. *Families, systems & health : the journal of collaborative family healthcare* 2010, **28**(2):146-160.

69. Hawley ST, Janz NK, Lillie SE, Friese CR, Griggs JJ, Graff JJ, Hamilton AS, Jain S, Katz SJ: **Perceptions of care coordination in a population-based sample of diverse breast cancer patients**. *Patient education and counseling* 2010, **81 Suppl**:S34-40.

70. Velikova G, Keding A, Harley C, Cocks K, Booth L, Smith AB, Wright P, Selby PJ, Brown JM: **Patients report improvements in continuity of care when quality of life assessments are used routinely in oncology practice: secondary outcomes of a randomised controlled trial**. *European journal of cancer* 2010, **46**(13):2381-2388.

71. Wilberforce M, Harrington V, Brand C, Tucker S, Abendstern M, Challis D: **Towards integrated community mental health teams for older people in England: progress and new insights**. *Int J Geriatr Psychiatry* 2011, **26**(3):221-228.

72. **The Joint Commission Patient-Centered Medical Home (PCMH) Self-Assessment Tool web site** [<http://www.jointcommission.org/joint_commission_primary_care_medical_home_self-assessment_tool___/>]

73. Guevara JP, Greenbaum PE, Shera D, Shea JA, Bauer L, Schwarz DF: **Development and psychometric assessment of the collaborative care for attention-deficit disorders scale**. *Ambulatory pediatrics : the official journal of the Ambulatory Pediatric Association* 2008, **8**(1):18-24.

74. Forrest CB, Glade GB, Starfield B, Baker AE, Kang M, Reid RJ: **Gatekeeping and referral of children and adolescents to specialty care**. *Pediatrics* 1999, **104**(1 Pt 1):28-34.

75. **CAHPS Surveys and Tools to Advance Patient-Centered Care web site** [https://[www.cahps.ahrq.gov/Surveys-Guidance/CG/PCMH.aspx](http://www.cahps.ahrq.gov/Surveys-Guidance/CG/PCMH.aspx)]

76. Seid M, Varni JW, Bermudez LO, Zivkovic M, Far MD, Nelson M, Kurtin PS: **Parents' Perceptions of Primary Care: measuring parents' experiences of pediatric primary care quality**. *Pediatrics* 2001, **108**(2):264-270.

77. Jean-Pierre P, Fiscella K, Freund KM, Clark J, Darnell J, Holden A, Post D, Patierno SR, Winters PC, Patient Navigation Research Program G: **Structural and reliability analysis of a patient satisfaction with cancer-related care measure: a multisite patient navigation research program study**. *Cancer* 2011, **117**(4):854-861.

78. Chen AY, Schrager SM, Mangione-Smith R: **Quality measures for primary care of complex pediatric patients**. *Pediatrics* 2012, **129**(3):433-445.

79. Chodosh J, Pearson ML, Connor KI, Vassar SD, Kaisey M, Lee ML, Vickrey BG: **A dementia care management intervention: which components improve quality?** *American Journal of Managed Care* 2012, **18**(2):85-94.

80. Jesmin S, Thind A, Sarma S: **Does team-based primary health care improve patients' perception of outcomes? Evidence from the 2007-08 Canadian Survey of Experiences with Primary Health**. *Health Policy* 2012, **105**(1):71-83.

81. McDonald K, Schultz E, Albin L, Pineda N, Lonhart J, Sundaram V, Smith-Spangler C, Brustrum J, Malcolm E: **Care Coordination Atlas Version 3 (Prepared by Stanford University under subcontract to Battelle on Contract No. 290-04-0020)**. Rockville, MD: Agency for Healthcare Research and Quality; November 2010.
